# Complete mitogenome of tea green leafhopper, *Empoasca onukii* (Hemiptera: Cicadellidae) from Anshun, Guizhou Province in China

**DOI:** 10.1080/23802359.2017.1398616

**Published:** 2017-11-09

**Authors:** Jian-Hong Liu, Cui-Ying Sun, Ju Long, Jian-Jun Guo

**Affiliations:** Key Laboratory of Insect Informative System and Resource Development and Utilization of Guizhou Province, School of Agriculture, Anshun University, Anshun, China

**Keywords:** *Empoasca onukii*, mitogenome, molecular phylogeny

## Abstract

Tea green leafhopper, *Empoasca onukii* (Hemiptera: Cicadellidae), is one of the most serious pest insects in tea plantations in Asia. In this study, Mitogenome of this species was assembled with high coverage using Illumina sequencing data and the 15,167 bp length was acquired. The base composition is 38.1% for A, 11.2% for C, 10.5% for G, and 40.2% for T. The mitogenome includes 13 protein-coding genes, 22 transfer RNA, and two ribosomal RNA genes. The phylogeny showed that it is closely related to *E. vitis* with high bootstrap value supported. The complete mitogenome of *E. onukii* can provide essential DNA molecular data for further phylogenetic and evolutionary analysis.

Tea plant, *Camellia sinensis*, is native to Asia, the Indian subcontinent and it is today cultivated in tropical and subtropical climate regions across the world. Tea green leafhopper, *Empoasca onukii* Massuda (Hemiptera: Cicadellidae), is one of the most serious pest insects in tea plantations in Asia (Qin et al. 2015; Shi et al. 2017). Nymph and adult of *E. onukii* sucks sap from young tea shoots and leaves and female adults lay their eggs into the branches, causing damage called hopperburn (Muraleedharan and Chen 1997). Occurrence generations for this species were dependent on climatic conditions and 9–17 generations were detected every year in different areas in China. The peaks for population normally occur in June and September, resulting in serious loss of tea production (Shi et al. 2014, 2015). Considering the pest’s economic importance in agriculture, it is essential to identify the complete mitochondrial genome and the related phylogeny.

Samples were collected from tea plants by sweep net from Pingba district (26.41°N, 106.25°E) of Anshun city, Guizhou Province of southwest China during tea green leafhopper occurrence. Specimens were preserved in absolute alcohol and deposited in Key Laboratory of Insect Information Systems and Resource Development and Utilization of Guizhou Province, School of Agriculture, Anshun University, Anshun, China. Genomic DNA was extracted following the manufacturer’s instruction in the DNeasy Blood and Tissue kit (Qiagen, Hilden, Germany) for insect tissue. Sequencing was conducted using the Illumina HiSeq platform (San Diego, CA). The sequence was preliminarily aligned within the CLUSTAL X program in BioEdit software (Thompson et al. 1997; Hall 1999). Protein-coding genes (PCGs), rRNA, and tRNA genes were predicted by using MITOS tools (Bernt et al. 2013).

The complete mitogenome of *P. dives* is 15,167 bp long in size (GenBank MG190360). The base composition is 38.1% for A, 11.2% for C, 10.5% for G, and 40.2% for T. Complete mitogenome of *E. onukii* contains 13 PCGs, 22 transfer RNA genes, two ribosomal RNA genes and a major non-coding region known as the CR (control region). J-strand codes nine PCGs (NAD2-3, NAD6, COX1-3, CYTB, ATP6, and ATP8) and 14 tRNAs, while N-strand codes four PCGs (NAD1, NAD4, NAD4l, and NAD5), eight tRNAs, and two rRNAs (16S rRNA and 12S rRNA). Gene arrangement of mitogenome for *E. onukii* is identical to the most common type of the putative ancestor of insects (Boore 1999; Cameron 2014).

To validate the phylogenetic position, the mitogenomes of 19 species in suborder Auchenorrhyncha of Hemiptera order and an outgroup *Drosophila melanogaster* (Diptera: Drosophilidae) were clustered together with *E. onukii* to construct ML tree by using the maximum likelihood method based on the Tamura-Nei model in MEGA 7 software (Tamura and Nei 1993; Kumar et al. 2016). The tree inferred from 500 replicates was taken to represent the phylogeny of the species analysed in this study (Felsenstein 1985). Result indicated that *E. onukii* was closely related to *E. vitis* (Hemiptera: Cicadellidae) with high bootstrap value supported (Figure 1). Furthermore, three clades were correctly identified as assigned and monophyletic family Cicadellidae, Delphacidae, and Cercopidae with high bootstrap confidence (Figure 1). In conclusion, the mitochondrial genome of *E. onukii* deduced in present study can provide essential DNA molecular data for further phylogenetic and evolutionary analysis.

**Figure 1.**
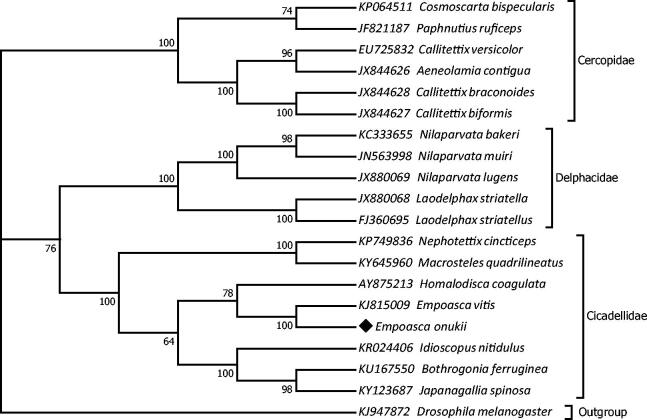
Molecular phylogeny of tea green leafhopper, *Empoasca onukii* (GenBank MG190360) and the related species in order Hemiptera based on complete mitogenome. Phylogenic tree is constructed by maximum likelihood method with 500 bootstrap replicates. Genbank accession number for tree construction is listed before the scientific name of species. The position of *E. onukii* is marked in solid diamond shape.
